# Prostaglandin D_2_ effects and DP_1_/DP_2_ receptor distribution in guinea pig urinary bladder out‐flow region

**DOI:** 10.1111/jcmm.12959

**Published:** 2016-09-23

**Authors:** Na N. Guan, Karl Svennersten, Petra J. de Verdier, N. Peter Wiklund, Lars E. Gustafsson

**Affiliations:** ^1^ Department of Physiology and PharmacologyKarolinska InstitutetStockholmSweden; ^2^ Department of Molecular Medicine and SurgeryKarolinska InstitutetStockholmSweden; ^3^ Present address: Department of Laboratory MedicineKarolinska Institutet at Karolinska University Hospital HuddingeS‐141 86StockholmSweden

**Keywords:** prostaglandins, smooth muscle, urinary tract, trigone, proximal urethra, internal urethral sphincter, PGD_2_, DP_1_, DP_2_

## Abstract

The proximal urethra and urinary bladder trigone play important roles in continence. We have previously shown that PGD_2_ is released from guinea pig bladder urothelium/suburothelium and can inhibit detrusor contractile responses. We presently wished to investigate PGD_2_ actions in guinea pig out‐flow region and the distribution of DP_1_/DP_2_ receptors. The effects of PGD_2_ on urothelium‐intact trigone and proximal urethra contractility were studied in organ bath experiments. Expression of DP_1_/DP_2_ receptor proteins was analysed by western blot. Immunohistochemistry was used to identify distribution of DP_1_/DP_2_ receptors. PGD_2_ in a dose‐dependent manner inhibited trigone contractions induced by electrical field stimulation (EFS) and inhibited spontaneous contractions of the proximal urethra. PGD_2_ was equally (trigone) or slightly less potent (urethra) compared with PGE_2_. Expression of DP_1_ and DP_2_ receptors was found in male guinea pig bladder trigone, neck and proximal urethra. In the trigone and proximal urethra, DP_1_ receptors were found on the membrane of smooth muscle cells and weak immunoreactivty was observed in the urothelium. DP_2_ receptors were distributed more widespread, weakly and evenly in the urothelium and smooth muscles. Inhibitory effects by PGD_2_ on motor activity of guinea pig trigone and proximal urethra are consistent with finding DP_1_ and DP_2_ receptors located in the urothelium and smooth muscle cells of the trigone and proximal urethra, and PGD_2_ may therefore be a modulator of the bladder out‐flow region, possibly having a function in regulation of micturition and a role in overactive bladder syndrome.

## Introduction

The lower urinary tract (LUT) consists of the urinary bladder and the urethra with the functions of urine storage and periodic urination. Continence and micturition involve a balance between detrusor activity and urethral sphincter closure. Bladder filling and voiding are controlled by a complex pattern of afferent and efferent signalling in parasympathetic, sympathetic and somatic pathways 1, 2. In addition to neural control, other mediators including prostaglandins are involved in this process. Expression of cyclo‐oxygenase in the urinary bladder has been found 3. Production of prostaglandins locally within the urinary tract in human and other species has also been well‐studied. Disturbed release of inhibitory and excitatory factors could result in incontinence and even break the balance between filling and voiding.

In the LUT, the bladder trigone is a smooth region in the base of urinary bladder within the two ureteral orifices and the internal urethral orifice which has different embryological origins from the rest part of the bladder. The majority of vessels and nerves of the bladder enter and concentrate in the trigone making it very sensitive to expansion 1, 4. Continence is maintained by three major urethral sphincter mechanisms, and which differ between male and female, namely the internal urethral sphincter (IUS), the external urethral sphincter (EUS) and the periurethral levator ani muscles. The existence and morphology of an IUS is a debated issue. Some studies suggest that smooth muscles in the bladder neck and proximal urethra are a continuation of the bladder body detrusor 5, 6. Other results suggested that the structure and autonomic innervation of smooth muscle in the bladder neck and proximal urethra differ from that in the bladder detrusor 7. The EUS consists of skeletal muscle with a thin layer of smooth muscle in the area of the membranous urethra which is under voluntary neuronal control. Although both are composed by striated muscle, the EUS muscles and levator ani muscles are morphologically distinct 8, 9.

Prostaglandin D_2_ is an important lipid mediator that exerts its biological functions *via* the G protein‐coupled receptors prostaglandin D receptor type 1(DP_1_) and prostaglandin D receptor type 2 (DP_2_), the later also known as chemoattractant homologous receptor expressed on Th2 cells (CRTH2). We have previously shown that PGD_2_ and PGE_2_ were released from guinea pig urinary bladder and that PGD_2_ inhibited induced bladder detrusor contractions 10. The effects of PGE_2_ on the trigone and proximal urethra has been studied for decades. Andersson and colleagues showed that PGE_1_ and PGE_2_ relaxed pre‐contracted human circularly cut urethral rings 11. In another study, PGE_2_ was shown to relax the pre‐contracted trigone and longitudinally cut human and pig urethra 12. Similar results were also found in other species, *i.e*. PGE_2_ relaxed the pre‐contraction of circularly cut hamster and longitudinally cut dog urethra 13, 14. One study of cat urethra found that PGE_2_ contracted the longitudinal urethra strips but relaxed the circular urethra muscle 15. In rabbit and dog trigone, PGE_2_ enhanced the tone and increased spontaneous activities 14, 16. Whether PGD_2_ is involved in the regulation of trigone and urethra motility remains unknown. In the human, expression of functional DP receptors was found in corpus cavernosum smooth muscle 17. In guinea pig urinary bladder dome, DP_1_ receptors was found in the smooth muscle and urothelium with a dominant localization to smooth muscle membranes, DP_2_ was also found on the bladder wall 18. Data concerning the expression of DP_1_ and DP_2_ receptors in the proximal urethra and trigone regions have not been reported.

In the present study, we examined the effects of PGD_2_ and PGE_2_ on male guinea pig trigone and proximal urethra and report on the efficacy of PGD_2_ and PGE_2_ in these tissues. We describe the expression and distribution of DP_1_ and DP_2_ receptors in trigone and proximal urethra with respect to their distribution in both urothelium and muscle components.

## Materials and methods

### Tissue preparation

All experiments were approved by the local animal ethics committee (Dnr N178/11). Male albino guinea pigs weighing 500–750 g were anaesthetized with midazolam 1 mg/kg + sodium pentobarbital 120 mg/kg and exsanguinated. The urinary bladder and proximal urethra were removed *en bloc*. Seminal vesicles, deferent ducts, coagulating gland ducts and ejaculatory ducts were removed at duct openings. The trigone was dissected by locating the urethra and ureter openings. A trigone strip about 7 × 2 mm was made from each guinea pig and with the urothelium intact. The IUS ring from the level of bladder neck to above duct openings of the proximal urethra was dissected with intact urothelium was opened and cut into 1–2 strips for organ bath experiments. All tissue strips were tied at both ends with thin cotton threads and equilibrated in 5.5 ml organs bath containing Tyrode's solution (136.9 mM NaCl, 4.8 mM KCl, 23.8 mM NaHCO_3_, 0.5 mM MgCl_2_, 0.4 mM NaH_2_PO_4_, 2.5 mM CaCl_2_, and 5.5 mM glucose) and aerated with 5% CO_2_ in O_2_ at 37°C.

### Organ bath experiments

After 30 min. equilibration, one end of the tissue was connected to an isometric transducer and the other end to a hook at the bottom of the bath. Tissues were carefully washed with Tyrode's three times. The initial resting tension of the trigone and urethra strips was adjusted to 5 mN. Proximal urethra strips were left unstimulated to record the spontaneous contractions. When stable tension developed, trigone strips were electrically stimulated by means of two platinum electrodes on the walls of the organ baths (50 V, 3 Hz, 0.2 msec., 15 pulses at 60 sec. intervals). The evoked contractions were recorded with a computerized acquisition system (MP100; Biopac Systems, Goleta, CA, USA). When stable contractions were recorded, diclofenac 10^−6^ M was given to the trigone tissues to inhibit the production of endogenous prostaglandins. After 10 min. incubation with diclofenac, the tissues were washed and diclofenac 10^−6^ M was reapplied to trigone strips throughout the experiment.

#### PGD_2_ and PGE_2_ effects on guinea pig trigone contractions induced by EFS

PGD_2_ and PGE_2_ were added to the tissues cumulatively in log increments from 10^−9^ to 10^−6^ M. Each dose was applied for 10 min. Control contraction amplitudes were measured before application of PGD_2_ and PGE_2_. Contractile response at 10 min. at every dose of PGD_2_ and PGE_2_ were measured and compared with control amplitude. Log concentration‐response curves were constructed.

#### PGD_2_ and PGE_2_ effects on guinea pig proximal urethra spontaneous activities

After regular spontaneous contractions were developed, PGD_2_ and PGE_2_ were given to the urethra strips cumulatively in half‐log or log increments from 10^−9^ to 10^−6^ M at 8–10 min. intervals in the absence of diclofenac since cyclo‐oxygenase inhibitors were reported to inhibit spontaneous contractions in the urinary tract 19, 20, 21. The waveform area of spontaneous contractions was measured by integration before and at 8 min. in the presence of PGD_2_ and PGE_2_ and expressed as area per min. Log concentration‐response curves were constructed.

#### Solvent effects on tissue contractions

The corresponding amount of ethanol used to dissolve PGE_2_ and PGD_2_ was applied cumulatively in log increments to trigone and urethral tissues without any compound. The final concentration of ethanol in each organ bath was less or equal to 0.1%.

### Western Blot

#### Tissue preparation

Male guinea pigs were anesthetized as above and the abdominal aorta was flushed distally with 30–40 ml warm saline to achieve blood‐free tissues. The bladder trigone, neck and proximal urethra were dissected and isolated apart. For protein extraction, each mg wet tissue was subjected to 20 μl of lysis buffer (pH 7.6) containing 20 mM Hepes, 150 mM NaCl, 5 mM ethylenediaminetetraacetic acid, 25 mM KF, 1 mM sodium orthovanadate, 0.5% Triton X‐100, 20% glycerol and 1% protease inhibitor cocktail (Sigma‐Aldrich, St. Louis, MO, USA). Tissues were homogenized using an Ultra‐Turrax for 2 min. and then homogenized for 4 min. in a Dounce glass homogenizer. Lysates were centrifuged at 13,000 × g gravity for 20 min. at +4°C. Protein content of the supernatant was determined with the Bradford protein assay (Bio‐Rad Laboratories, Hercules, CA, USA). 7 μg of protein was loaded onto 8–16% SDS Pierce Protein Gel (Thermo Scientific, Rockford, IL, USA) and separated by electrophoresis. Proteins were transferred onto PVDF membranes using dry blot/iBLOT according to the manufacturer's instructions (Invitrogen, Carlsbad, CA, USA). Membranes were blocked for one hour with 5% skim milk dissolved in PBS‐T (PBS, 0.1% Tween 20). Membranes were probed overnight with rabbit anti‐human DP_1_ receptor C‐terminal antibody (1:1000, ab99446; Abcam, Cambridge, UK) or rabbit anti‐human DP_2_ (CRTH2) receptor antibody (1:2000, NBP1‐76755; Novus Biologicals, Abingdon, UK) diluted in PBS‐Tween 20 with 5% skim milk. HRP‐conjugated goat anti‐rabbit secondary antibodies (1:10,000; Thermo Scientific) and Supersignal West Femto Chemiluminescent Substrate (Thermo Scientific) were used to detect protein signal on autoradiographs (Kodak X‐Omat 2000 processor; Kodak, New York, NY, USA).

### Fluorescence immunohistochemistry and microscopy

#### Tissue preparation

Male guinea pigs were anaesthetized and perfused as above. The urinary bladder with short ureter remains and proximal urethra were taken *en bloc* and cleaned from connective tissues. The bladder was flushed of urine before fixation which was by immersion in ice‐cold 4% paraformaldehyde 0.1 M phosphate buffer fixative solution for 4 hrs at 4°C. After fixation, tissues were cryoprotected by incubation in 0.1 M phosphate buffer with 30% sucrose solution for 16–20 hrs at 4°C. The trigone and urethra were then dissected from the bladder. The proximal urethra was cut transversely into several ring segments. Small pieces of tissue were covered with Neg‐50^™^ (Thermo Scientific) and quickly frozen in liquid nitrogen cooled isopentane and stored at −80°C for later cryostat sectioning. 10 μm cryostat sections of vertical trigone and transverse urethra were mounted on gelatin coated slides.

#### Fluorescence immunohistochemistry

Cryostat sections were blocked in blocking buffer with PBS (pH 7.2) containing 0.5% Triton X‐100 and 5% normal bovine serum for 20 min. at room temperature. Sections were either labelled with the rabbit anti‐human DP_1_ receptor antibody (1:250, ab99446; Abcam) or labelled with the rabbit anti‐human DP_2_ receptor antibody (1:300, NBP1‐76755; Novus Biologicals). All the antibodies were diluted in blocking buffer. The sequential control sections were treated with blocking buffer. Cryostat sections labelled with antibodies were incubated overnight at 4°C. Before secondary antibody treatment, the sections were rinsed three times for 5 min. in PBS. Then, sections were incubated for 1 hr with secondary antibodies at room temperature. Counterstaining of smooth muscle actin, F‐actin and nuclei was made by monoclonal anti‐α‐smooth muscle actin antibody (1:500, C6198; Sigma‐Aldrich), phalloidin (1:500, P5282; Invitrogen) and Hoechst33258 (1:2000, 94403; Sigma‐Aldrich), respectively. The secondary antibodies used in this study were: donkey anti‐rabbit antibody labelled with Alexa Fluor 568 (1:500, A‐10042; Invitrogen) and donkey anti‐rabbit antibody labelled with Alexa Fluor 488 (1:500, A‐11055; Invitrogen). After 1 hr incubation, sections were rinsed three times in PBS for 5 min. in the dark. The sections were then mounted in S3023 medium with anti‐fading agent (Dako, Glostrup, Denmark) and covered with coverslips.

#### Microscopy

All immunolabelled sections were observed under an Axioplan 2 imaging fluorescence microscope (Carl Zeiss MicroImaging GmbH, Jena, Germany) equipped with FITC (Chroma 41001), TRITC (Chroma 41002a) and DAPI (Chroma #31000) filters. Sections were photographed with a Nikon D7000 digital camera using NKRemote software (version 2.2; Breeze Systems, Camberley, UK) for camera control with 12‐bit image acquisition followed by subsequent background subtraction and contrast enhancement in ImageJ (NIH open source).

### Chemicals and data analysis

PGE_2_ and PGD_2_ were generous gifts from Professor Ernst H Oliw (Uppsala Universitet). Diclofenac, atropine, tetrodotoxin and D‐tubocurarine were from Sigma‐Aldrich. All the data are presented as mean ± S.E.M. PGE_2_ and PGD_2_ log dose–response curves were fitted by a 4‐parameter sigmoidal curve model using Prism 5 (GraphPad Software Inc., La Jolla, CA, USA) to estimate the IC50 of PGE_2_ and PGD_2_.

## Results

### Spontaneous activity in strips from trigone and proximal urethra and their responses to nerve stimulation by EFS

**Figure 1:**
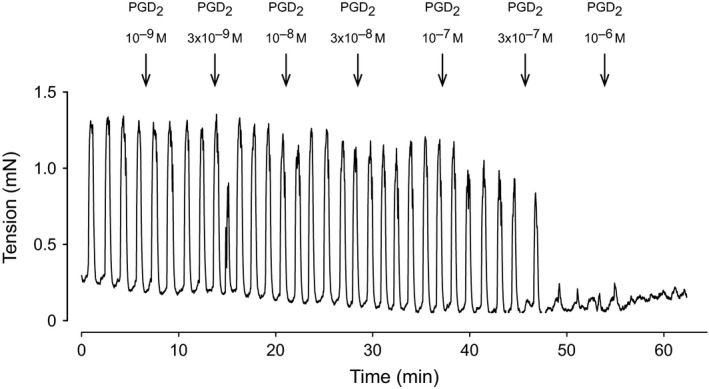
Original experimental recording of spontaneous contractions in an isolated male guinea pig proximal urethra preparation with urothelium intact, and the effect of prostaglandin D_2_ (PGD_2_). Arrows at 8–10 min. intervals indicate the cumulative administration of PGD_2_ in half‐log increments from 10^−9^ to 10^−6^ M final bath concentration. Isometric recording.

*In vitro*, unstimulated urothelium intact guinea pig urinary bladder trigone strips showed irregular, fast and fast‐relaxing (3–4 sec.) spontaneous contractions with a frequency of 4.71 ± 0.74 contractions per min (*n* = 7). When the trigone strips were electrically stimulated (50 V, 3 Hz, 0.2 msec., 15 pulses at 60 sec. intervals), monophasic and reproducible post‐stimulation contractile responses were observed. These were nerve‐mediated as indicated by their sensitivity to tetrodotoxin. Muscle strips from the level of bladder neck had no spontaneous activity and did not respond to nerve stimulation. Strips of proximal urethra taken below the bladder neck and above the duct openings, representing the major region of the IUS, showed regular, slow and long‐lasting (30–40 sec.) spontaneous contractions (Fig. 1). This region did not respond to EFS. Urethra strips from below duct openings and above the bulbourethral glands showed similar spontaneous contractions as in the IUS region, but exhibiting lower frequency. Individual separate fast contractions were seen when EFS (50 V, 3 Hz, 0.2 msec., 15 pulses) was applied to this region. The spontaneous contractions in trigone and proximal urethra were not modified by atropine or D‐tubocurarine.

### Effects of prostaglandin E_2_ and D_2_ on trigone contractile responses to EFS

**Figure 2:**
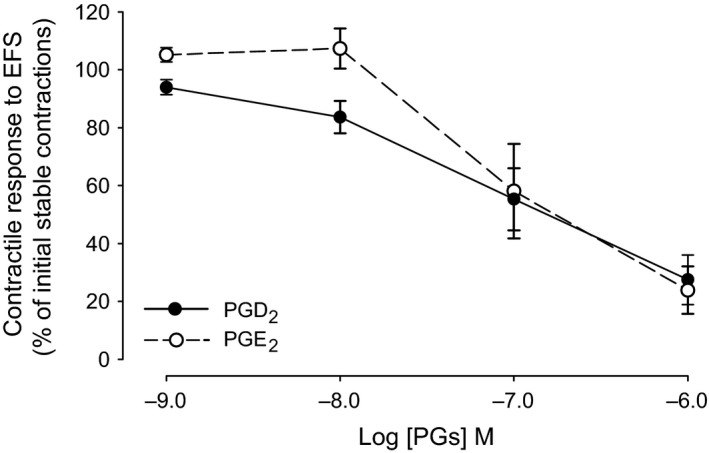
Dose–response curves of PGD_2_ and PGE_2_ (cumulatively from 10^−9^ to 10^−6^ M) on contractile responses to EFS in guinea pig urothelium intact trigone strips. Tissues were subjected to electrical field stimulation at 50 V, 3 Hz, 0.2 msec., 15 pulses at 60 sec. intervals. Mean amplitudes of stable trigone contraction before any compound were defined as 100% control. Data are presented as mean ± S.E.M. *n* = 5 for PGE_2_ and *n* = 6 for PGD_2_, n denotes number of animals.

Reproducible repeated contractile responses of isolated trigone were induced by EFS as above. After treatment with diclofenac (10^−6^ M), PGE_2_ or PGD_2_ was applied cumulatively from 10^−9^ to 10^−6^ M. PGE_2_ and PGD_2_ inhibited the EFS‐induced contractions in a dose dependent manner. In some experiments, low concentrations of PGE_2_ (10^−9^ to 10^−8^ M) elicited a weak enhancement of contractile responses, whereas higher concentrations (above 10^−8^ M) always elicited inhibition. Dose–response curves (Fig. 2) gave an estimated pIC_50_ for PGE_2_ of 7.05 ± 0.27 (*n* = 5) and an estimated pIC_50_ value for PGD_2_ of 7.08 ± 0.27 (*n* = 6). The corresponding amounts of ethanol used to dissolve PGE_2_ and PGD_2_ had no effect on EFS contractions when applied to the trigone strips.

### Effects of prostaglandin E_2_ and D_2_ on spontaneous contractions in proximal urethra

**Figure 3:**
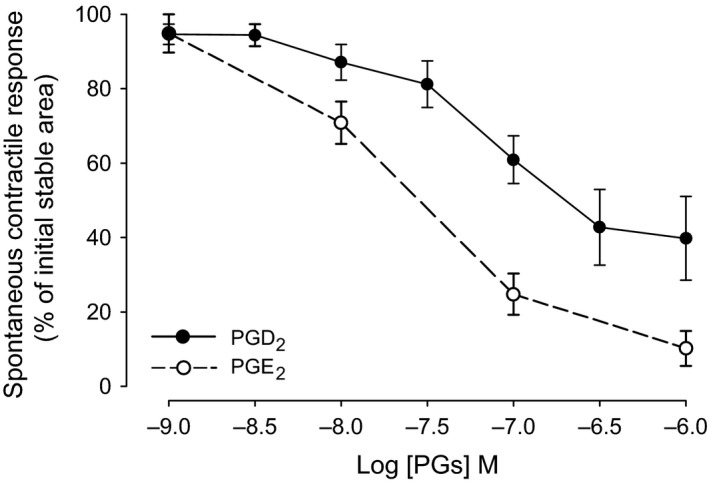
Dose–response curves of PGD_2_ and PGE_2_ (cumulatively from 10^−9^ to 10^−6^ M) on spontaneous contractions in guinea pig urothelium intact proximal urethra strips. Stable integrated waveform areas of proximal urethra spontaneous contractions, before applying any compound, were used as 100% control. Plots were made by comparing areas per min in the absence after each concentration of PGD_2_ or PGE_2_ application. Data presented as mean ± S.E.M., *n* = 7 for PGE_2_ and *n* = 14 for PGD_2_ where n denotes number of animals.

Urethral strips from below the neck and above the duct openings, which is the IUS region exhibited regular spontaneous contractions that were unaffected by atropine 5 × 10^−7^ M or D‐tubocurarine 10^−6^ M as mentioned above. When PGE_2_ or PGD_2_ 10^−9^ to 10^−6^ M was given cumulatively to the IUS, inhibition of spontaneous contractions were seen (Fig. 1). In the IUS region, with a PGD_2_ concentration greater than 3 × 10^−7^ M, there was little or no effect by PGD_2_ on the resting muscle tone. In contrast, urethral strips from below the openings, corresponding to part of the EUS, showed a strong tonic contraction with 10^−7^ M PGD_2_. Dose–response curves for the inhibitory effect by the prostaglandins on the IUS are shown in Figure 3. The estimated pIC_50_ was 7.65 ± 0.17 (*n* = 7) for PGE_2_ and 7.09 ± 0.22 (*n* = 14) for PGD_2_. Ethanol at the corresponding concentrations as used to dissolve PGE_2_ and PGD_2_ did not modify the IUS spontaneous contractions.

### Expression of prostaglandin DP_1_ and DP_2_ receptors in male guinea pig trigone, bladder neck and proximal urethra

**Figure 4:**
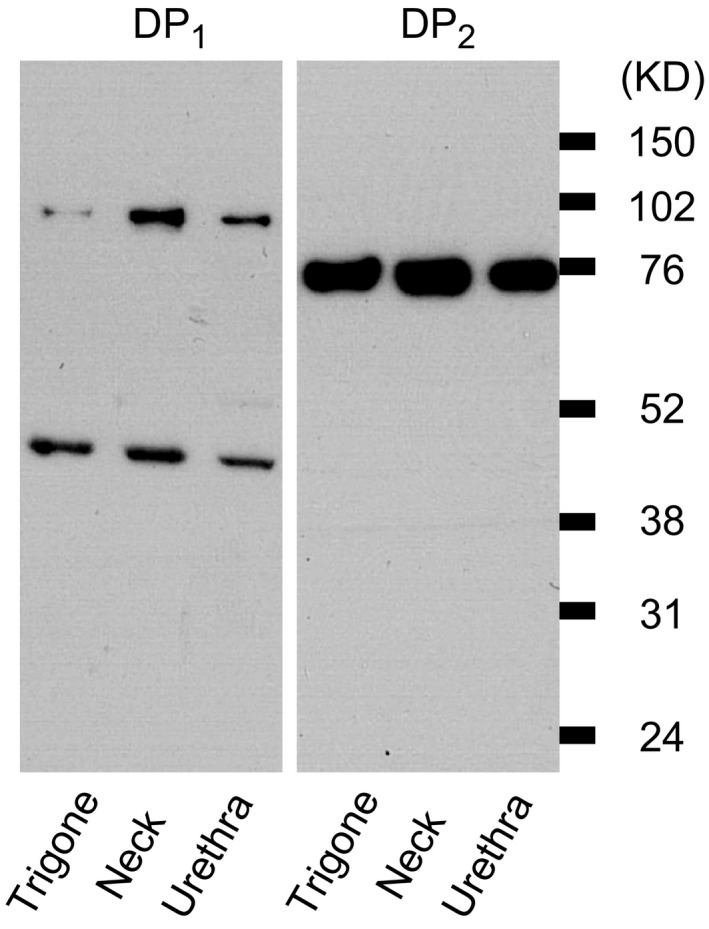
Detection of DP_1_ and DP_2_ receptor protein in male guinea pig trigone, bladder neck and proximal urethra by Western blot. The three lanes of panel DP_1_ and 3 lanes of panel DP_2_ were loaded with the same amount of total protein from trigone, neck and urethra tissue extracts. After electrophoresis separation, panel DP_1_ was subjected to a rabbit anti‐human DP_1_ receptor antibody and panel DP_2_ was treated with a rabbit anti‐human DP_2_ (CRTH2) receptor antibody. Markers on the right indicates positions of molecular‐weight protein standards.

The expression of DP_1_ and DP_2_ (CRTH2) receptor proteins in the guinea pig trigone, bladder neck and proximal urethra was examined by Western blot. Tissue extracts, containing the whole layers of both muscle and urothelium from these regions were exposed to DP_1_ and DP_2_ antibodies and which were the same antibodies used in the immunohistochemistry. As shown in Figure 4, the male guinea pig trigone, neck and urethra expressed significant levels of DP_1_ and DP_2_ proteins. In Figure 4, DP_1_ panel, two groups of protein bands at, respectively, the predicted molecular weight for DP_1_ (40 kD) and at around 95 kD were seen. This result fits the data provided by the manufacturer in tests with the antibody on different cell lines. In Figure 4, DP_2_ panel, only one group of DP_2_ protein bands was observed at around 75 kD. The predicted band location for DP_2_ is 40 kD, the reason for observing an increased size of the protein bands is likely a reported post‐translational modification, *e.g*. phosphorylation, glycosylation *etc*. 22.

### Distribution of DP_1_ and DP_2_ receptors in male guinea pig trigone

**Figure 5:**
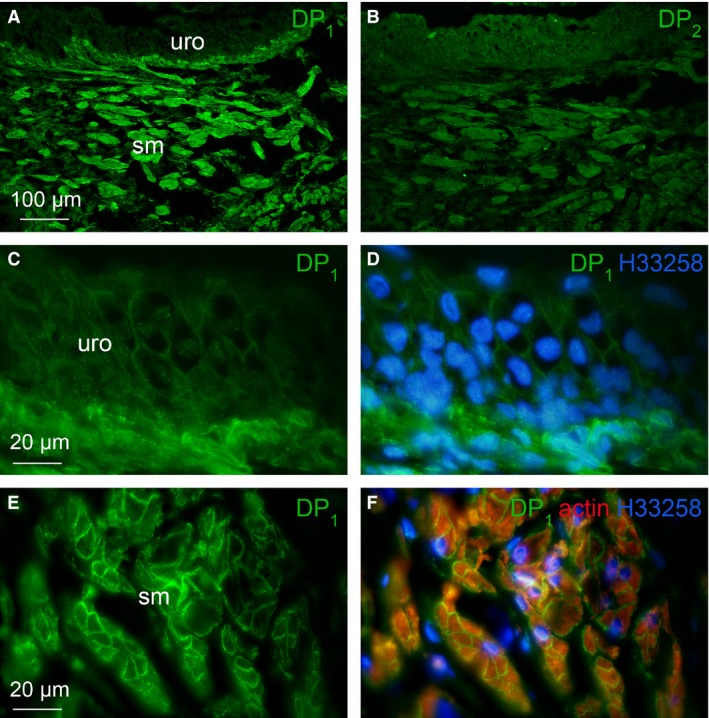
Cryosections of guinea pig trigone showing by fluorescence immunohistochemistry the distribution of DP_1_ and DP_2_ receptors in the urothelium and smooth muscle layers. (**A**) It displays the label for anti‐DP_1_ (green) and (**B**) the label for anti‐DP_2_ (green) and were sequential sections from the same trigone tissue. (**C**–**F**) Theses are details of section in **A**), showing distribution of DP_1_ receptor in the urothelium (**C** and **D**) and in smooth muscle bundles (**E** and **F**). Muscle actin was counterstained with F‐actin phalloidin reagent (red) visualized in **F**, superimposed on the image from **E**. Nuclei were counterstained with Hoechst 33258 (blue) as visualized in **D** and **F**. ‘uro’ indicates urothelium layer, ‘sm’ indicates smooth muscle layer. Scale bars indicate 100 μm in **A** and **B**, 20 μm in **C**–**F**.

Fluorescence immunohistochemistry results from sequential vertical sections of the trigone with transitional urothelium (uro) and part of the smooth muscle (sm) layers are shown in Figure 5A and B. Immunoreactivity to DP_1_ and DP_2_ receptors was seen throughout the trigone urothelium and smooth muscle. DP_1_ receptors were more prominent in the smooth muscle layer while DP_2_ receptors were more evenly distributed in the urothelium and smooth muscle layers but not as strong and localized as DP_1_ receptors in the muscle cells. Figure 5C and D show the details of DP_1_ receptor distribution in the urothelium of the trigone. The border between urothelium and suburothelium was heavily stained for DP_1_ receptor with localization to the cells immediately below the membrane but only faint fluorescence was seen in the urothelium layer, with a predominant localization to urothelium cell membranes. Figure 5E and F indicate the details of DP_1_ receptor distribution in the smooth muscle bundles. As shown in Figure 5F, fluorescence with the DP_1_ receptor antibody was found surrounding the red fluorescence for muscle actin, indicating localization of DP_1_ receptor mainly in the membranes of smooth muscle cells. The blue fluorescence in Figure 5D and F denotes the counterstaining of the nuclei. Negative control sections (*i.e*. not exposed to primary antibody, only secondary antibodies) showed no staining for DP_1_ or DP_2_ receptors.

### Distribution of DP_1_ and DP_2_ receptors in male guinea pig proximal urethra

**Figure 6:**
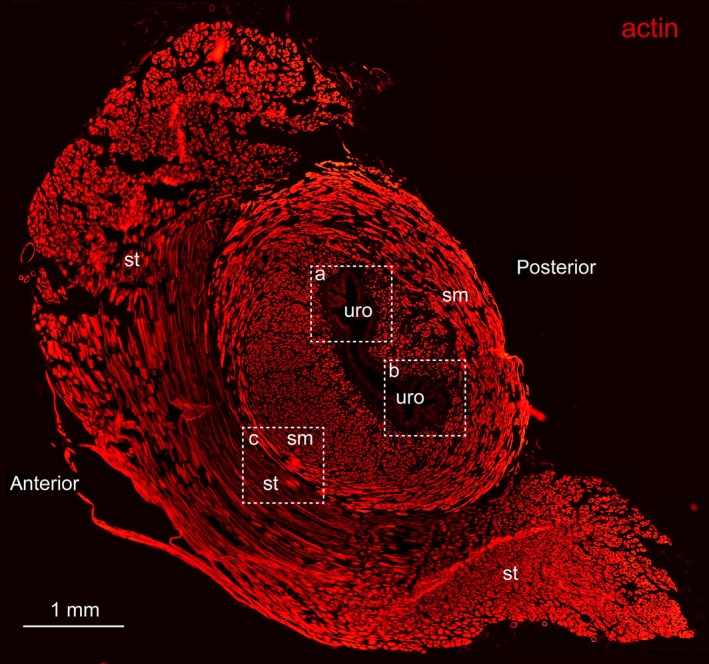
Transverse section of male guinea pig proximal urethra obtained above the duct openings and labelled with F‐actin phalloidin reagent visualized in red. ‘uro’ indicates urothelium layer, ‘sm’ indicates smooth muscle layer. ‘st’ indicates striated muscle. Box ‘a’ and box ‘b’ show the corresponding urothelium regions as in Figure 7 and Figure 8. Box ‘c’ indicates the border region between smooth muscle and striated muscle as in Figure 9.

The morphology of the male guinea pig urethra differs depending on the position at which sections are taken. In this study, we focused on the proximal urethra with the IUS. Figure 6 shows a transverse section from the IUS region labelled with F‐actin phalloidin reagent to reveal the structure of muscle. The oval shape of urethra can be seen in the centre, containing regions of both circular and longitudinal smooth muscle (sm) which was surrounded by semi‐circular striated muscle bundles. In the urethra, a layer of urothelium (uro) was faintly stained for F‐actin. Between smooth muscle (sm) and striated muscle (st), some smooth muscle bundles insert into striated muscle bundles (Fig. 6 box ‘c’, Fig. 9 C2 and C4). The smooth muscle component became thinner along the length of the urethra. Distally of the duct openings only a diamond‐ring shaped thin layer of smooth muscle was found with a thickness between 65 to 230 μm (data not shown).

**Figure 7:**
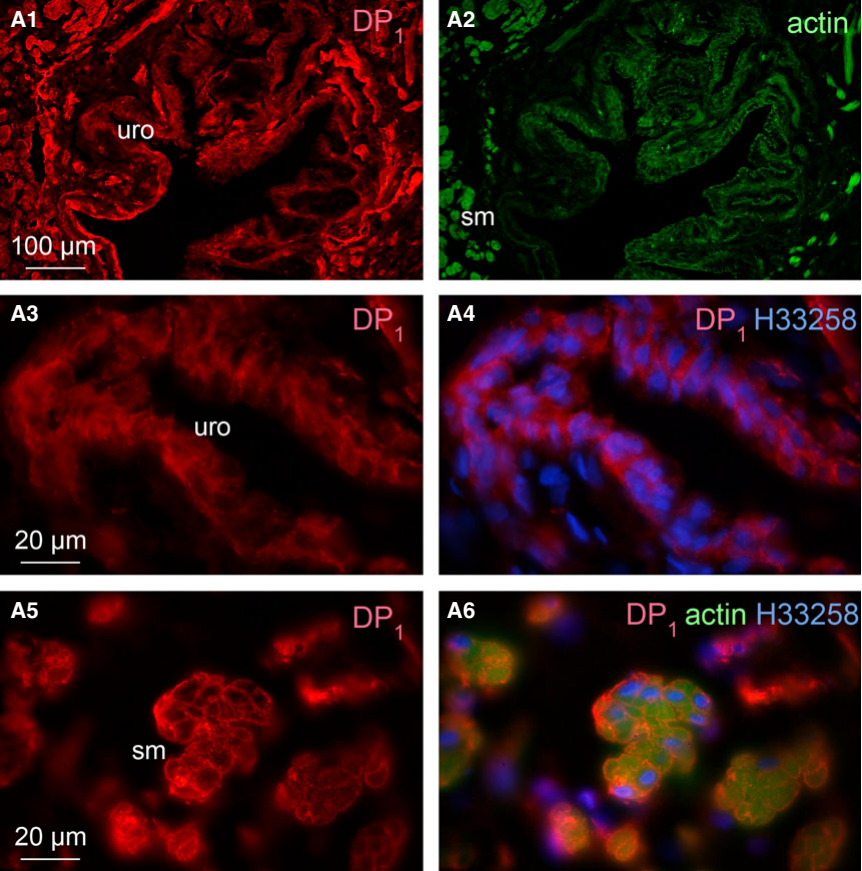
Fluorescence immunohistochemistry in cryosection of male guinea pig proximal urethra, adjacent to the section in Figure 6, showing at higher magnifications details of the area corresponding to box ‘a’ in Figure 6. Distribution of DP_1_ receptors in the urothelium and smooth muscle was visualized by anti‐DP_1_ (red) antibody in **A1** and **A3–A6**. Actin was visualized by staining with F‐actin phalloidin reagent (green). Nuclei were counterstained with Hoechst33258 (blue). **A2** corresponds to **A1** but visualized for the label for actin. **A3** and **A4** are higher magnifications of the urothelium, **A4** being a superimposition with the nuclear stain on the image in **A3. A5** and **A6** are higher magnifications of the smooth muscle below the suburothelium, **A6** being a superimposition on **A5** with the combination of the visualization of actin (green) and nuclei (blue). ‘uro’ indicates urothelium layer, ‘sm’ indicates smooth muscle layer. Scale bars indicate 100 μm in **A1** and **A2**, 20 μm from **A3** to **A6**.

**Figure 8:**
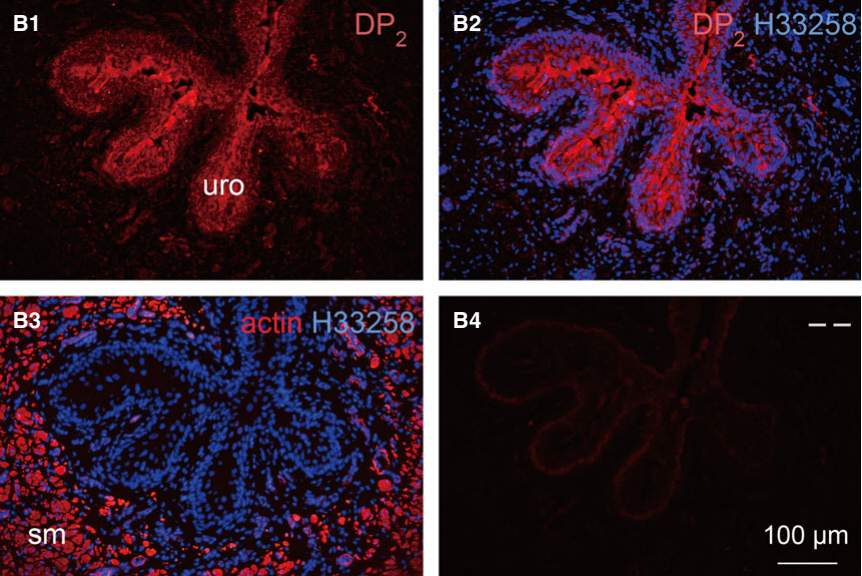
Fluorescence immunohistochemistry in cryosections of male guinea pig proximal urethra, adjacent to the section in Figure 6, showing at higher magnifications details of the area corresponding to box ‘b’ in Figure 6. Distribution of DP_2_ receptors in the urothelium and smooth muscle was visualized by label for anti‐DP_2_ (red) antibody in **B1** and **B2**, where **B2** is a superimposition of the nuclear stain with Hoechst 33258 (blue) on image in **A1**. Staining for DP_2_ receptors was very faint in the smooth muscle. Sequential section **B3** was labelled with anti‐α‐actin (red) showing the corresponding smooth muscle positions in **B1**. Sequential section **B4** was secondary antibody control for DP_2_ receptor antibody. ‘uro’ indicates urothelium layer, ‘sm’ indicates smooth muscle layer. Scale bar is 100 μm in all sections.

In Figure 7 (higher magnification of adjacent section of box ‘a’ in Fig. 6), immunoreactivity to DP_1_ receptor was seen distributed in the proximal urethra urothelium (Fig. 7A3, A4) and smooth muscle (Fig. 7A5, A6). The distribution of DP_1_ receptor was similar as in the trigone where fluorescence of DP_1_ receptor antibody was seen more heavily stained in the smooth muscle than in the urothelium (Fig. 7A1, A3, A5). The smooth muscle components of the section in Figure 7A1 were visualized with F‐actin phalloidin reagent as shown in Figure 7A2. The distribution of DP_1_ receptor was found on the membranes of smooth muscle cells surrounding the green fluorescence for muscle actin (Fig. 7A6). DP_2_ receptor immunoreactivity was also seen distributed in the proximal urethra urothelium and smooth muscle as seen in Figure 8 (higher magnification of adjacent section of box ‘b’ in Fig. 6). The urothelium exhibited much stronger fluorescence for DP_2_ receptor compared with the smooth muscle (Fig. 8B1, B2 and B3). The blue fluorescence in all figures was the counterstaining of the nuclei. Negative control sections showed no staining for DP_2_ receptors (Fig. 8B4).

**Figure 9:**
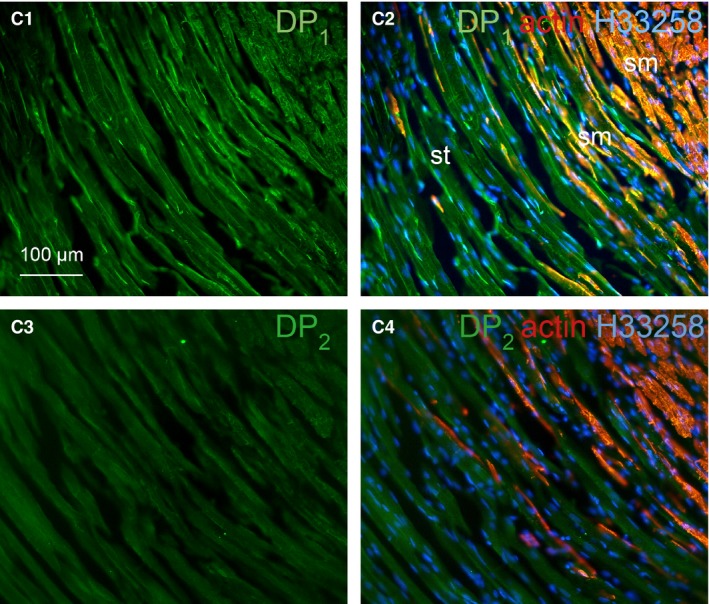
Fluorescence immunohistochemistry in cryosections of male guinea pig proximal urethra, adjacent to the section in Figure 6, showing at higher magnifications details of the area corresponding to box ‘c’ in Figure 6. Distribution of DP_1_ and DP_2_ receptors in the smooth muscle and striated muscle, at the border between these two types of muscle, is demonstrated. Section in **C1** and **C2** (**C2** being a superimposition on image in **C1**) was labelled with anti‐DP_1_ (green), section in **C3** and **C4** (**C4** being a superimposition on image in **C3**) was labelled with anti‐DP_2_ (green). Section **C2** and **C4** were counterstained with anti‐α‐smooth muscle actin (red) and Hoechst33258 nuclei stain (blue). Yellow fluorescence represents the co‐localization of DP_1_ receptors and anti‐α‐smooth muscle actin. ‘sm’ indicates smooth muscle layer. ‘st’ indicates striated muscle layer. Scale bar is 100 μm for all sections.

The border between smooth muscle (sm) and striated muscle (st) in male guinea pig proximal urethra is shown in Figure 9 (higher magnification of adjacent section of box ‘c’ in Fig. 6). Longitudinal and circular smooth muscle layers were labelled red with anti‐α‐smooth muscle actin antibody as shown Figure 9C2 and C3. DP_1_ and DP_2_ receptors were found both in smooth muscle and striated muscle components, but DP_2_ receptor was stained at a much lower degree. Merged image of muscle anti‐DP_1_ receptor (green) and anti‐α‐smooth muscle actin (red) shows strong yellow fluorescence indicating co‐localization of DP_1_ receptor on the smooth muscle bundles (Fig. 9C2). The distribution of DP_1_ receptor was more profound in the smooth muscle compared with striated muscle (Fig. 9C1).

## Discussion

The major novel findings in the present study are that, in male guinea pig trigone and proximal urethra PGD_2_ can exert inhibitory influence on smooth muscle contractile responses induce by EFS and on spontaneous contractions. DP_1_ and DP_2_ receptors are found expressed in the trigone and proximal urethra. In the bladder trigone, DP_1_ receptors are markedly located on the suburothelium layer and smooth muscle cells membranes similar as in the proximal urethra. DP_2_ receptors are found weakly and evenly on the urothelium/suburothelium layers and also in the smooth muscle.

A previous *in vitro* study using human bladder strips found no effect of PGD_2_ up to 3 × 10^−7^ M on resting tension and was contractile at higher concentrations 23. Our previous work showed that PGD_2_ was produced in guinea pig urinary bladder in a urothelium‐dependent fashion and exerted its inhibitory effects on EFS induced contractions 10. This effect was shown to be *via* DP_1_ receptors localized on the detrusor membrane 18. Trigone and proximal urethra IUS are the important regions involved in controlling micturition. The trigone musculature is dually innervated by both the sympathetic and parasympathetic systems. Contraction of the trigone reduces the resistance of the bladder neck, thus facilitating urination. The contraction also pulls the orifices of the ureter ends to the bladder neck resulting in a strong increase in the uretero‐vesical resistance and serves as an important element for the valve function 24. When the bladder is filled with low volumes of urine, continence can be maintained by passive resistive elements of the urethral outlet. As bladder volume increases, use of a sympathetic reflex is necessary to maintain continence. The EUS can be voluntarily contracted to counter abrupt elevations of intravesical pressure 25. From our previous and present results it is possible to suggest that during the filling stage, PGD_2_ together with other relaxing mediators such as PGE_2_ is produced by the urothelium/suburothelium to relax the underlying smooth muscle. When the bladder reaches a threshold volume, an emptying process is triggered where PGD_2_ together with other mediators might reduce the amplitude of initial detrusor muscle contractions, but also by relaxation of the internal sphincter might facilitate entry of urine into the proximal urethra. Passage of urine into the proximal urethra is a signal in the initiation of micturition 26.

We observed distinct regional inherent activity and responses to EFS along the proximal urethra. An earlier study measuring the urethral pressure profile of the male feline urethra using a silicone rubber catheter with pressure transducers showed increased urethra pressure from proximal to distal with several peaks at the region of prostatic urethra, bulbourethra and penile urethra 27. In our study, we found that bladder neck was relatively quiescent and did not respond to mild EFS (50 V, 3 Hz, 0.2 msec., 15 pulses at 60 sec. intervals) nor to have spontaneous activity. Since we failed to observe any EFS‐induced contractions in the bladder neck, we cannot conclude on whether prostaglandins play a role in regulating nerve‐induced movements in this region. We believe that the region between the bladder neck and duct openings where we observed regular inherent contractions forms part of the IUS, since we by histochemistry identified an IUS consisting of an inner longitudinal layer and an outer circular layer in the region between neck and duct openings. The spontaneous electrical and mechanical activity of this region contributes to overall muscular tone 24, 28.

The validity of our histochemical results on DP_1_ and DP_2_ receptor distribution is supported by our Western blot data and by results with similar antibody in a study by Zhang *et al*. who showed that DP_1_ and DP_2_ receptors are present in guinea pig oesophageal nodose ganglia by immunostaining and Western blot with similar antibody, and by RT‐PCR 29. Presently, in male guinea pig trigone and proximal urethra, we found both DP_1_ and DP_2_ receptors located in the urothelium and smooth muscle and that DP_1_ receptors were prominent on the membranes of smooth muscle cells, in agreement with our previous study on bladder detrusor 18. PGD_2_ might therefore be suggested to directly bind to the receptors located on the smooth muscle, tentatively regulating the contractility by modulating muscle cAMP level since this is a known mechanism in the PGD_2_ inhibitory action on smooth muscle 30.

Some limitations of this study include the fact that it was an *in vitro* design using male guinea pig trigone and proximal urethra strips. *In vivo* experiments dealing with the whole LUT with intact neural system will be necessary to determine the exact functional implications of our results. Studies on female urethra should also be carried out in the future.

## Conclusion

The present study suggests an inhibitory influence of PGD_2_ on the guinea pig trigone and proximal urethra and is consistent with the expression and distribution of DP_1_ and DP_2_ receptors in these regions. PGD_2_ may therefore be a modulator of the bladder out‐flow region, possibly having a function in regulation of micturition and a role in overactive bladder syndrome. The information is of value for our further understanding of the LUT physiology and provides a foundation for future studies on the human out‐flow region.

## Author contribution

NG conceived and designed the study and performed all the organ bath experiments after discussion with LG. NG and PdV performed the Western blots and NG and KS performed the immunohistochemistry experiments which were evaluated together with LG and PW. NG compiled the data and made all the draft figures which were finalized together with LG. NG drafted the first manuscript which was reviewed and revised by all authors.

## Conflict of interest

The authors confirm that there are no conflicts of interest.
